# Identification of quinazoline compounds as novel potent inhibitors of Wnt/β-catenin signaling in colorectal cancer cells

**DOI:** 10.18632/oncotarget.7019

**Published:** 2016-01-25

**Authors:** Yonghe Li, Wenyan Lu, Surendra K. Saini, Omar Moukha-Chafiq, Vibha Pathak, Subramaniam Ananthan

**Affiliations:** ^1^ Drug Discovery Division, Southern Research Institute, Birmingham, AL 35205, United States of America

**Keywords:** Wnt signaling, Wnt inhibitor, quinazoline, colorectal cancer, drug discovery

## Abstract

The Wnt/β-catenin signaling pathway is critical for the initiation and progression of most colon cancers, and has emerged as one of the most promising targets for colorectal cancer chemoprevention and treatment. In this study, we have discovered a structurally related series of quinazolines as potent inhibitors of Wnt/β-catenin signaling in colorectal cancer cells harboring mutations in *CTNNB1* or *APC*. We showed that the quinazoline leads suppressed Wnt/β-catenin signaling without altering the level of β-catenin protein in colorectal cancer cells, suggesting that they act on the downstream elements of the pathway. Moreover, the quinazoline leads displayed potent anticancer activities with IC_50_ values between 4.9 and 17.4 μM in colorectal cancer cells. Importantly, we also found that a structurally related quinazoline lacking inhibitory effect on Wnt/β-catenin signaling was unable to suppress colorectal cancer cell proliferation. Together, these results suggest that the quinazoline lead compounds identified in this study have therapeutic potential for the prevention and treatment of colorectal cancer.

## INTRODUCTION

Wnt/β-catenin signaling is important for cancer progression, including tumor initiation, tumor growth, cell death, and metastasis [1-5]. β-Catenin is the central player in signal transduction of this pathway. In the absence of Wnt ligands, β-catenin levels are efficiently regulated by a supramolecular complex containing adenomatous polyposis coli (APC), axin, and glycogen synthetase kinase 3β (GSK3β). This complex promotes β-catenin phosphorylation and subsequent β-catenin degradation. Several components of the Wnt/β-catenin pathway have been identified as oncogenes or tumor suppressors (showing gain-of-function or loss-of-function mutations, respectively) in many types of human cancers [1-3]. Mutations in these genes are most evident in colorectal cancers. About 80% of all colorectal cancers contain loss-of function mutations in the tumor suppressor gene APC. Gain-of-function mutations in the oncogene *CTNNB1* (β-catenin encoding gene) are present in approximately 10% of the colorectal cancers. The consequence of either *APC* inactivation or *CTNNB1* activation mutations is the failure of proper β-catenin degradation leading to its cytosolic accumulation. The β-catenin then translocates into the nucleus where it interacts with T-cell factor/lymphoid enhancing factor (TCF/LEF) to induce the expression of downstream target genes [6, 7]. It is well established that aberrant activation of Wnt/β-catenin signaling is a necessary initiating event in the genesis of most colorectal cancers [6-8], and that the Wnt/β-catenin pathway has emerged as one of the most promising targets for colorectal cancer chemoprevention and treatment [1, 3, 9-11].

Among heterocyclic compounds, libraries of compounds possessing the quinazoline template in particular have attracted considerable attention in lead discovery efforts. Contributing factors for their desirability include: (a) facile synthetic access, (b) lead-like and drug-like attributes and (c) opportunities that the template provides for molecular manipulations for lead optimizations. The introduction of quinazolines such as lapatinib and gefitinib as kinase inhibitory drugs for cancer treatment exemplifies recent successes in optimization of quinazoline lead compounds into drugs [12-15].

In our efforts to discover novel inhibitors of Wnt/β-catenin signaling, we have identified a structurally related series of quinazolines as potent inhibitors in colorectal cancer cells harboring mutations in *CTNNB1* or *APC*. Although a few quinazoline analogs have been reported in the literature as inhibitors of the Wnt/β-catenin signaling pathway [16-19], the set of compounds that we have identified is structurally distinct. Moreover, the quinazoline leads displayed potent anticancer activities with IC_50_ values between 4.9 and 17.4 μM for colorectal cancer cells, and the IC_50_ values are comparable to their inhibitory activities on Wnt/β-catenin signaling in colorectal cancer cells.

## RESULTS

### Quinazoline leads suppress Wnt/β-catenin signaling in colorectal cancer cells and inhibit cell proliferation

**Figure 1:**
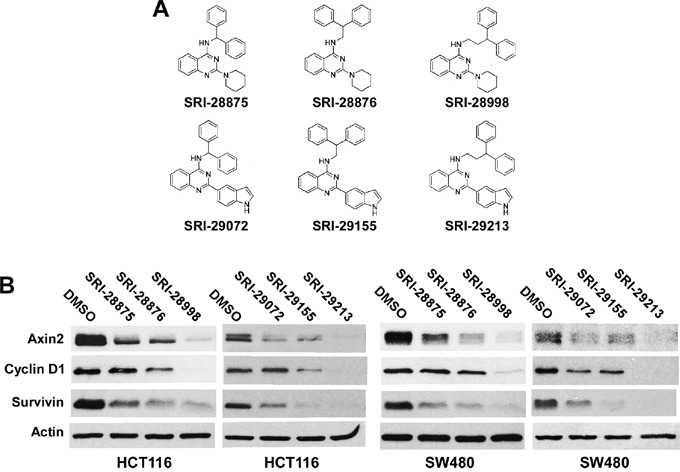
Effects of the six lead compounds on Wnt/β-catenin signaling in colorectal cancer cells **A.** Structures of the six lead quinazolines. **B.** Cancer cells in 6-well plates were treated with test compounds at 10 μM (HCT116) or 20 μM (SW480) for 24 h. The levels of axin2, cyclin D1 and survivin were examined by Western blotting. All the samples were also probed with anti-actin antibody to verify equal loading.

In support of lead discovery efforts on various biological targets, we synthesized a library of ∼ 500 compounds containing the quinazoline template. From this library of quinazolines, we screened a set of 40 compounds using the Super8XTOPFlash Wnt reporter assay in colorectal cancer HCT116 cells (Supplementary Table S1). Interestingly, results from this screen led to the identification of two sets of structurally related quinazolines as inhibitors of Wnt/β-catenin signaling (Figure 1A and Table 1). Axin2 is a specific transcriptional target of Wnt/β-catenin signaling, and the expression level of axin2 is the signature of the activation of the Wnt/β-catenin pathway [20-23]. In addition, cyclin D1 and survivin have been identified as two key transcriptional targets of the Wnt/β-catenin pathway, and are critical for cancer cell proliferation and cell death [24-27]. Additional studies with these compounds revealed that they suppressed the expression of Wnt/β-catenin signaling targets axin2, cyclin D1 and survivin in both colorectal cancer HCT116 cells (harboring a *CTNNB1* mutation) and SW480 cells (harboring an *APC* mutation) (Figure 1B), and displayed potent anticancer activities with IC_50_ values between 4.9 and 17.4 μM for HCT116, SW480 and SW620 cells (Table 1). The IC_50_ values of these quinazoline inhibitors are comparable to their inhibitory activities on Wnt/β-catenin in colorectal cancer cells (Table 1), suggesting that the anticancer activities of the quinazoline leads are associated with their inhibitory effects on Wnt/β-catenin signaling in colorectal cancer cells.

**Table 1:** Activity data on the lead compounds

ID	MWT	CLogP^a^	PSA^a^	Wnt reporter activity in HCT116 cells, IC_50_ (μM)	Cell viability, IC_50_ (μM)		
					HCT116	SW480	SW620
SRI-28875	395	6.2	39.9	14.7	11.9	17.4	13.6
SRI-28876	409	6.9	39.9	6.6	7.9	13.5	10.2
SRI-28998	423	7.3	39.9	6.0	5.2	7.0	8.2
SRI-29072	427	6.7	48.7	22.3	8.8	12.3	6.0
SRI-29155	441	7.5	48.7	9.7	8.5	8.9	9.2
SRI-29213	455	7.8	48.7	8.9	4.9	5.8	7.1
SRI-31230	332	5.5	39.9	12.7	6.2	5.9	6.7

a CLogP and Polar Surface Area (PSA) were calculated using ChemBioDraw Ultra 2010.

### The presence of diaryl group in SRI-28876 is not essential for its inhibitory effect on Wnt/β-catenin signaling in colorectal cancer cells

**Figure 2:**
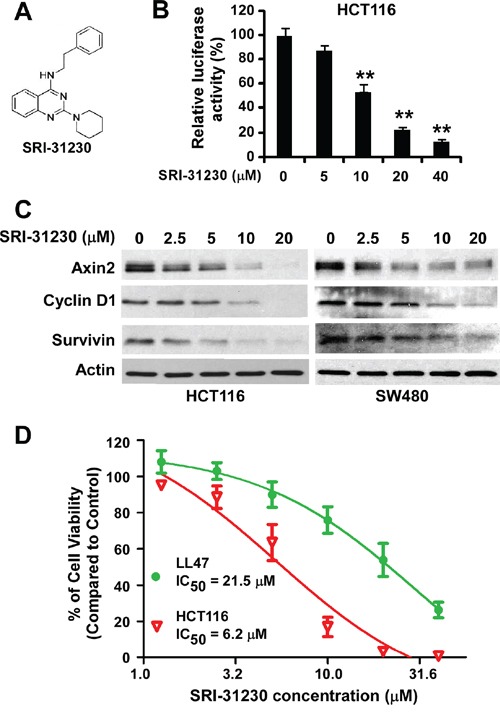
Effects of the lead compound SRI-31230 on Wnt/β-catenin signaling in colorectal cancer cells **A.** The structure of SRI-31230. **B.** Cancer cells in 24-well plates were transiently transfected with Super8XTOPFlash construct and β-galactosidase-expressing vector in each well. After being incubated for 24 h, cells were treated with SRI-31230 at the indicated concentrations for 24 h. The luciferase activity was then measured 24 h later with normalization to the activity of the β-galactosidase. Values are averages of three determinations with the SD indicated by error bars. *P < 0.05, **P < 0.01 versus corresponding control cells without SRI-31230 treatment. **C.** Colorectal cancer HCT116 and SW480 cells in 6-well plates were treated with SRI-31230 at the indicated concentrations for 24 h. The levels of axin2, cyclin D1 and survivin were examined by Western blotting. All the samples were also probed with anti-actin antibody to verify equal loading. **D.** HCT116 and LL47 cells in 96-well plates were treated with SRI-31230 for 72 h. Cell viability was measured by the Cell TiterGlo assay. All the values are the average of triplicate determinations with the SD indicated by error bars.

Compound SRI-31230 is a structurally simplified analog of SRI-28876 (Figure 2A). We found that SRI-31230, similarly to SRI-28876, was able to suppress the Super8XTOPFlash Wnt reporter activity in HCT116 cells (Figure 2B), and inhibited the expression of Wnt/β-catenin signaling targets axin2, cyclin D1 and survivin in HCT116 cells and SW480 cells (Figure 2C). Moreover, SRI-31230 exhibited similar potent anticancer activity with IC_50_ values between 5.9 and 6.7 μM for HCT116, SW480 and SW620 cells (Figure 2D and Table 1). Together, these results indicate that the presence of diaryl group such as the diphenylethylamino moiety present in compound SRI-28876 may not be essential for the inhibitory effect of the quinazoline leads on Wnt/β-catenin signaling in colorectal cancer cells.

### SRI-31230 is able to selectively inhibit colorectal cancer cell proliferation

To further characterize our quinazoline lead SRI-31230, we performed cell proliferation/viability assay in normal lung fibroblast line LL47, and found that SRI-31230 was less cytotoxic against LL47 cells than colorectal cancer cells (Figure 2D), suggesting that SRI-31230 is able to selectively kill Wnt-dependent cancer cells.

### Quinazoline inhibitors suppress Wnt/β-catenin signaling by acting on the downstream elements of the pathway

**Figure 3:**
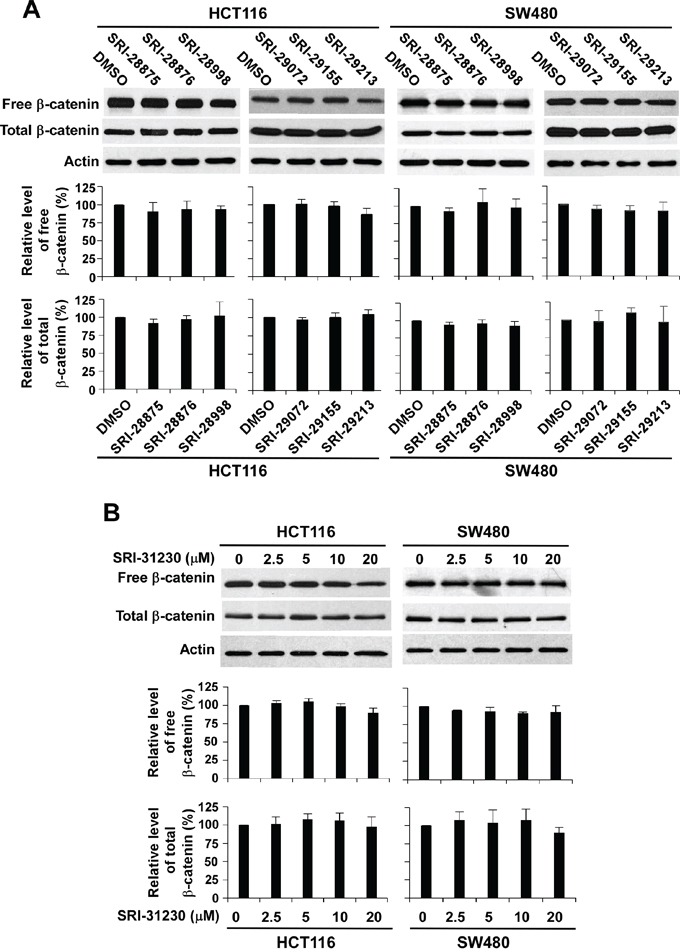
Effect of the seven lead compounds on levels of cytosolic free β-catenin and total β-catenin signaling in colorectal cancer cells **A.** Cancer cells in 6-well plates were treated with the test compounds at 10 μM (HCT116) or 20 μM (SW480) for 24 h. **B.** Cancer cells in 6-well plates were treated with SRI-31230 at the indicated concentrations for 24 h. The levels of cytosolic free β-catenin and total β-catenin signaling were examined by Western blotting. All the samples were also probed with anti-actin antibody to verify equal loading. The bands were quantified by densitometry using the Image J software. The intensity of the total cellular β-catenin bands was quantified relative to actin. The results represented in the histograms are shown as the mean ± SD and are the average of three independent experiments.

Uncomplexed cytosolic β-catenin (free β-catenin) can translocate to the cell nucleus to activate the transcription factors of the TCF/LEF family, leading to the transcription of Wnt target genes. To identify the site of action of the quinazoline leads in Wnt/β-catenin signaling, we determined the β-catenin levels after the treatment with the lead compounds. It was found that the levels of cytosolic β-catenin and total cellular β-catenin were not significantly changed in response to treatment with the six quinazoline leads at 20 μM for 24 h (Figure 3A) or SRI-31230 at 2.5 to 20 μM for 24 h (Figure 3B). These results indicate that these quinazoline inhibitors suppress Wnt/β-catenin signaling by acting on the downstream elements of the pathway.

### Inhibition of Wnt/β-catenin signaling by the quinazoline lead compounds are responsible for their anticancer activity

**Figure 4:**
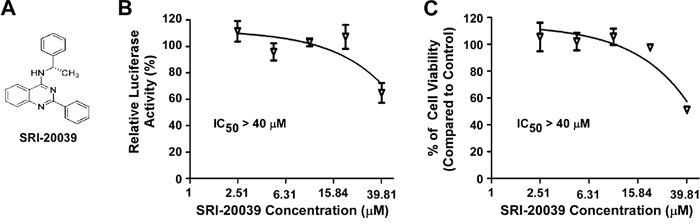
Effect of the compound SRI-20039 on Wnt/β-catenin signaling in colorectal cancer cells and cancer cell viability **A.** The structure of SRI-20039. **B.** HCT116 cells in 24-well plates were transiently transfected with Super8XTOPFlash construct and β-galactosidase-expressing vector in each well. After being incubated for 24 h, cells were treated with SRI-20039 at the indicated concentrations for 24 h. The luciferase activity was then measured 24 h later with normalization to the activity of the β-galactosidase. Values are averages of three determinations with the SD indicated by error bars. **C.** HCT116 cells in 96-well plates were treated with SRI-20039 for 72 h. Cell viability was measured by the Cell TiterGlo assay. All the values are the average of triplicate determinations with the SD indicated by error bars.

Compound SRI-20039 is a 2-phenylquinazoline derivative that was ineffective in blocking Wnt/β-catenin signaling in colorectal cancer cells. As a negative control, SRI-20039 displayed very weak activity against colorectal cancer cell proliferation (Figure 4). These results further suggest that inhibition of Wnt/β-catenin signaling by the quinazoline lead compounds are responsible for their anticancer activity.

## DISCUSSION

Aberrant activation of the Wnt/β-catenin signaling pathway is a necessary initiating event in the genesis of most colorectal cancers [6-8]. Accumulating evidence strongly suggests that novel inhibitors of Wnt/β-catenin signaling could emerge as chemotherapeutic agents for treatment of colorectal cancer [1, 3-5, 9-11]. Despite tremendous efforts in the past decade, most of the compounds reported in the literature including the clinical candidate LGK974 [28, 29], target upstream components of Wnt/β-catenin signaling [4, 5]. Such compounds may not be effective against colorectal cancers in which the Wnt/β-catenin pathway is deregulated by mutations in downstream components *AP*C and *CTNNB1*. Recent studies suggest that such targeting of downstream signaling could provide therapeutic advantages over targeting upstream signaling components [30, 31].

Among heterocyclic compounds, libraries of compounds possessing the quinazoline template in particular have attracted considerable attention in lead discovery efforts [12-15]. In the present study, we describe the discovery of a series of novel quinazolines possessing the drug-like quinazoline framework that inhibit the Wnt/β-catenin pathway in colorectal cancer cells. Our studies revealed that the quinazoline compounds repress Wnt/β-catenin signaling without altering the level of β-catenin protein in colorectal cancer cells harboring mutations in *CTNNB1* or *APC*, suggesting that they act on the downstream elements of the pathway. Importantly, we also found that a structurally related quinazoline lacking inhibitory effect on Wnt/β-catenin signaling was unable to suppress colorectal cancer cell proliferation, suggesting that the anticancer activity of the quinazoline lead compounds is associated with their inhibitory effect on Wnt/β-catenin signaling. However, additional studies are needed to dissect molecular mechanisms underlying the quinazoline-mediated inhibition of Wnt/β-catenin signaling in detail.

In summary, we have discovered a structurally related series of quinazolines as potent inhibitors of Wnt/β-catenin signaling in colorectal cancer cells. Of interest is the fact that structure–activity relationships (SAR) are discernible in the profile of this initial set of compounds, thus providing an opportunity to follow-up the SAR trend in a systematic way to enhance potency, selectivity, and drug-like characteristics in the future. The Wnt/β-catenin pathway is critical to uncontrolled cell proliferation in a number of tumor types, particularly colorectal cancer. Therefore, the quinazoline leads identified in the current effort are potentially promising candidates for development as novel chemotherapeutic agents for colorectal cancer.

## MATERIALS AND METHODS

### Materials

All of the test compounds were synthesized at Southern Research Institute. Details of the chemical synthesis will be published elsewhere. Plasmid pGST-E-cadherin was provided by Dr. Gail Johnson (University of Rochester, Rochester, NY). The Super8XTOPFlash luciferase construct was provided by Dr. Randall T. Moon (University of Washington, Seattle, WA). The β-galactosidase-expressing vector was obtained from Promega. Rabbit monoclonal anti-axin2 (#2151) was purchased from Cell Signaling Technology. Rabbit polyclonal anti-cyclin D1 (#04-221) was from EMD Millipore. Mouse monoclonal anti-survivin (D-8) (sc-17779) was from Santa Cruz Biotechnology. Mouse monoclonal anti-β-catenin (#61054) was from BD Biosciences. Mouse monoclonal anti-actin (#A2228) was from Sigma. Peroxidase labeled anti-mouse and anti-rabbit secondary antibodies and ECL system were purchased from Amersham Life Science. The luciferase and β-galactosidase assay systems were from Promega. Tissue culture media, fetal bovine serum (FBS), and plastic-ware were obtained from Life Technologies, Inc. Proteinase inhibitor cocktail Complete™ was obtained from Boehringer Mannheim.

### Cell culture

All cell lines were obtained from ATCC and grown under standard cell culture conditions at 37°C in a humidified atmosphere with 5% CO_2_. The cells were cultured in DMEM medium containing 10% of FBS, 2 mM of L-glutamine, 100 units/ml of penicillin, and 100 μg/ml of streptomycin.

### Western blotting

Colorectal cancer cells in 6-well plates were lysed in 0.5 ml of lysis buffer (phosphate-buffered saline containing 1% Triton X-100 and 1 mM PMSF) at 4°C for 10 min. Equal quantities of protein were subjected to SDS-PAGE under reducing conditions. Following transfer to immobilon-P transfer membrane, successive incubations with a primary antibody, and a horseradish peroxidase-conjugated secondary antibody were carried out for 60-120 min at room temperature. The immunoreactive proteins were then detected using the ECL system. Films showing immunoreactive bands were scanned by HP Scanjet 5590. Blots were quantitated by densitometry using Image J Software (NIH, Bethesda, MD, USA) and normalized to a housekeeper marker β-actin.

### Cytosolic free β-catenin analysis with GST-E-cadherin binding assay

The GST-E-cadherin binding assay was carried out exactly as previously described [32, 33]. Briefly, cells in six-well plates were lysed in 0.5 ml of lysis buffer at 4°C for 10 min, and extracts were clarified by centrifugation at 18,000×g for 2 min. One hundred micrograms of total cell extracts were incubated with Sepahrose beads bound to the GST-E-cadherin. The GST-E-cadherin Sepahrose beads were prepared as described [34]. After 1 h of incubation at 4°C, the Sepharose beads were collected by centrifugation at 10,000×g for 1 min, washed 2 times with lysis buffer containing 0.1% SDS and 0.5% bovine serum albumin and 2 times with PBS buffer, and boiled in SDS sample buffer containing β-mercaptoethanol. The supernatants were subjected to SDS–PAGE and Western blotting with the β-catenin antibody.

### Luciferase reporter assay for Wnt/β-catenin signaling

Colorectal cancer cells were plated into 24-well plates. After overnight culture, the cells were transiently transfected with the Super8XTOPFlash luciferase construct and β-galactosidase-expressing vector. After 24 h incubation, cells were treated with the test compounds at the indicated concentrations. Cells were then lysed 24 h later and both luciferase and β-galactosidase activities were determined. The luciferase activity was normalized to the β-galactosidase activity.

### Cell viability assay

Colorectal cancer cells were seeded into 96-well tissue culture treated microtiter plates at a density of 5000 cells/well. DMEM containing 10% FBS was used as assay media. After 24 h incubation, the cells were treated with the test compounds at the indicated concentrations for 72 h. Cell viability was measured by the Cell Titer Glo Assay, which is a luminescent assay that is an indicator of live cells as a function of metabolic activity and ATP content.

### Statistics

Statistical analyses were performed using Student's unpaired t-test. Data were presented as mean ± SD. Differences at P < 0.05 were considered statistically significant.

## SUPPLEMENTARY FIGURES AND TABLES




