# Supplementary material for: Identification of quinazoline compounds as novel potent inhibitors of Wnt/β-catenin signaling in colorectal cancer cells
Source: Oncotarget. 2016 Jan 25;7(10):11263–70. doi: 10.18632/oncotarget.7019 (PMC4905471; doi:10.18632/oncotarget.7019)
Supplement: Supplementary file 1 [file oncotarget-07-11263-s001.pdf]

SUPPLEMENTARY TABLE

Supplementary Table S1: Quinzaoline compounds evaluated in the Wnt signaling reporter assay

See Supplementary File 1
